# The Next-Generation CKD Heat Map: Hyperfiltration and eGFR Slope in the Cardiorenal Continuum

**DOI:** 10.1016/j.ekir.2026.106486

**Published:** 2026-03-17

**Authors:** Hiroshi Kataoka, Yusuke Ushio, Shun Manabe, Junichi Hoshino

**Affiliations:** 1 Department of Nephrology, Tokyo Women’s Medical University, Tokyo, Japan; 2 Department of Clinical Engineering, Faculty of Human Care at Makuhari, Tohto University, Chiba, Japan

Chronic kidney disease (CKD) has become one of the world’s fastest-rising causes of death, yet it is still often detected too late to allow meaningful prevention.S1 The 2023 CKD Prognosis Consortium heat map, later adopted into the 2024 Kidney Disease: Improving Global Outcomes (KDIGO) CKD guidelines, established the current global standard for risk stratification based on estimated glomerular filtration rate (eGFR) and albuminuria categories.^1^^,^^2^ This static, cross-classification approach transformed clinical communication by enabling clear visualization of adverse outcomes across 10 kidney and cardiovascular end points.

However, CKD risk assessment remains largely cross-sectional. The heat map captures severity at a single time point but does not explicitly incorporate physiologic or temporal dimensions of kidney function. An intriguing observation within the CKD Prognosis Consortium analysis is that individuals with very high eGFR (≥ 105 mL/min per 1.73 m^2^) combined with albuminuria may experience higher cardiovascular and mortality risk than patients with moderate CKD (eGFR: 30–44 ml/min per 1.73 m^2^).^1^^,^^2^ Rather than contradicting the heat-map framework, this pattern may reflect physiologic processes not explicitly represented in static staging models.

Here, we propose that 2 complementary dimensions—glomerular hyperfiltration and eGFR slope—provide a physiologically grounded framework for interpreting these observations across the cardiorenal continuum.^3^, ^4^, ^5^, ^6^, ^7^, ^8^^,^S2–S7

### Hyperfiltration as a Cardiorenal Load Phenotype

Hyperfiltration may represent one of the earliest detectable physiologic signals of filtration overload. The apparent paradox in the KDIGO heat map reflects a common misconception (Figure 1): high eGFR is often interpreted as preserved kidney function, although epidemiologic evidence suggests that very high filtration in the presence of albuminuria or cardiometabolic stress reflects systemic cardiorenal load rather than renal reserve.^1^^,^^6^^,^S2

**Figure 1:**
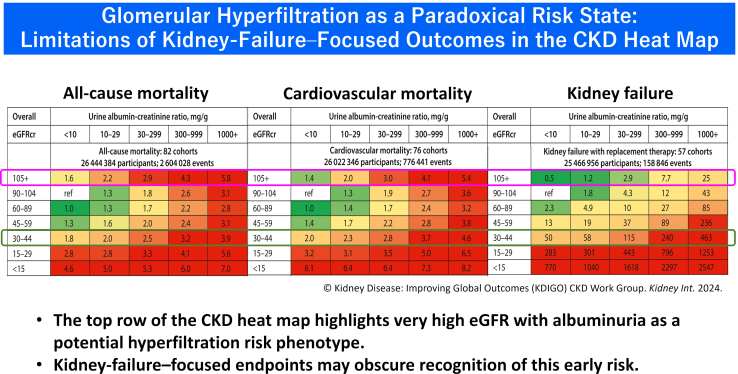
High eGFR unmasks a hidden high-risk phenotype (schematic). Individuals with very high eGFR (≥ 105 ml/min per 1.73 m^2^)—particularly when accompanied by albuminuria—experience all-cause and cardiovascular mortality exceeding that of moderate CKD. This pattern, preserved in Figure 5 of the KDIGO 2024 CKD guideline, suggests that hyperfiltration may reflect cardiorenal overload rather than preserved renal function and underscores potential limitations of purely static risk-staging frameworks. The heat-map structure is adapted from Figure 5 of the KDIGO 2024 CKD guideline. CKD, chronic kidney disease; eGFR, estimated glomerular filtration rate; eGFRcr, creatinine-based eGFR,

Hyperfiltration is therefore best understood not as a diagnostic category but as a clinical risk phenotype reflecting increased single-nephron workload within the cardiorenal continuum. In contemporary cardiometabolic disease, increased tubular transport demand and neurohormonal activation may drive adaptive, but potentially maladaptive, increases in single-nephron filtration. Hemodynamic adaptations driven by obesity, diabetes mellitus, insulin resistance, sympathetic activation, and tubular workload increase intraglomerular pressure and filtration demand, linking elevated filtration to structural nephron stress.^3^, ^4^, ^5^^,^^9^

Across multiple cohorts, hyperfiltration has been associated with accelerated kidney function decline, cardiovascular disease, and mortality.^1^^,^^3^^,^S2^,^S8–S12 Interpretation of elevated eGFR should therefore integrate clinical context, longitudinal trajectory, and age-related physiologic variation in filtration levels. Operational considerations for clinical interpretation are provided in the Supplementary Methods. Hyperfiltration may therefore represent one of the earliest physiologic signals of cardiorenal vulnerability along the lifetime filtration trajectory, providing a physiologic context for interpreting elevated eGFR within the broader cardiorenal continuum.

### Lifetime Trajectory: Hyperfiltration and Early Decline

Hyperfiltration may represent an early deviation in the lifetime eGFR trajectory. Increased nephron workload can produce an initially elevated filtration state followed by earlier decline (Figure 2a).^3^, ^4^, ^5^^,^S2

**Figure 2:**
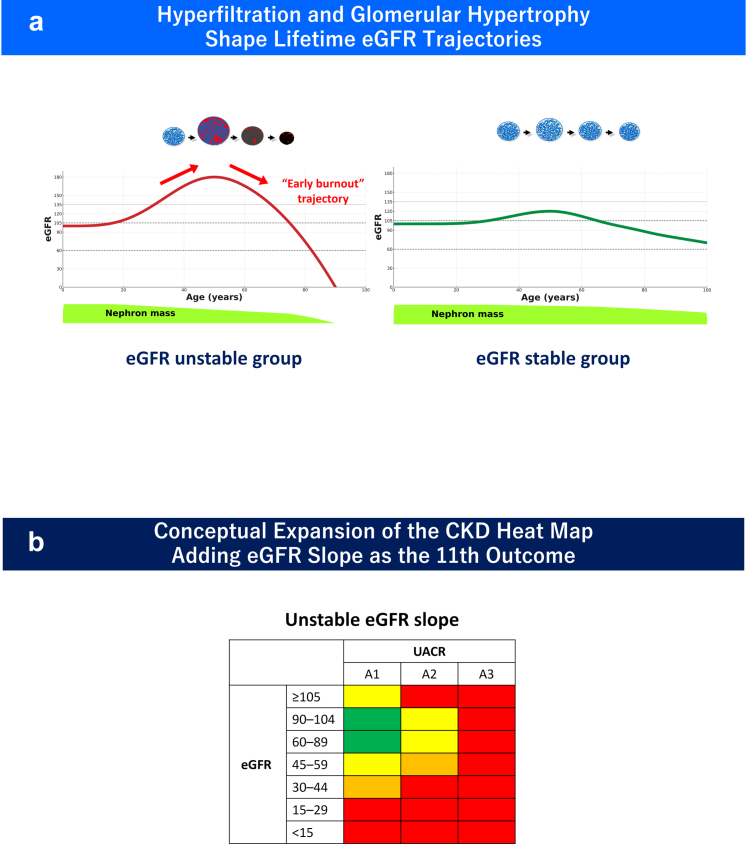
(a) Hyperfiltration and glomerular hypertrophy shape lifetime eGFR trajectories (schematic). Hyperfiltration initially elevates filtration through increased nephron workload but may shift the lifetime eGFR trajectory downward, resulting in earlier and steeper decline—a conceptual illustration of “early burnout” and filtration instability within the cardiorenal continuum. (b) eGFR slope as a dynamic “11th dimension” of kidney risk (schematic). Both steep decline and paradoxical increase in eGFR slope independently predict kidney failure, major cardiovascular events, and mortality—regardless of baseline eGFR category. Slope reflects filtration instability not captured by static eGFR–albuminuria staging and provides a dynamic marker linking filtration stress to downstream clinical outcomes. CKD, chronic kidney disease; eGFR, estimated glomerular filtration rate; UACR, urine albumin-to-creatinine ratio.

We refer to this conceptual trajectory as “early burnout,” in which filtration initially appears preserved or supranormal but subsequently declines more rapidly than expected for age. Extreme-value modeling suggests that supraphysiologic filtration represents a fragile and metabolically expensive physiologic state rather than enhanced renal reserve.^5^^,^S13 This trajectory-based interpretation links early filtration stress to later structural exhaustion across a continuous spectrum of kidney vulnerability.^3^^,^^9^^,^S4

Thus, hyperfiltration and kidney failure lie at opposite ends of a single spectrum: one represents early overload, the other represents terminal exhaustion. Especially in obesity-, diabetes-, and hypertension-related cardiorenal physiology,S9–S11^,^S14–S16 this perspective reframes early care, not as waiting for kidney-function decline to begin, but as recognizing early abnormalities in filtration dynamics.^3^^,^^5^ Importantly, this framework does not imply a single mechanism but integrates hemodynamic, metabolic, and structural processes that collectively precede the development of overt CKD. In this model, trajectory, not baseline eGFR, defines long-term risk.

### eGFR Slope: A Dynamic Marker of Filtration Instability

The 2024 KDIGO guideline relies primarily on static values of eGFR and albuminuria.^2^ Yet kidney risk unfolds over time, and instability is often more prognostic than severity.^3^^,^^5^

Longitudinal change in eGFR provides temporal insight into filtration dynamics that cross-sectional measures cannot capture. Rapid decline reflects structural injury characterized by microvascular rarefaction and tubular atrophy; whereas an unexpected increase in eGFR, an underrecognized risk state, may reflect hemodynamic hyperfiltration and has been associated with heart failure and mortality independent of baseline eGFR.^7,8,^S3–S7 Both declining and paradoxically increasing trajectories have been linked to kidney failure, major cardiovascular events, and all-cause mortality, independent of baseline eGFR or albuminuria.^8^^,^S3

Regulatory agencies (US Food and Drug Administration, European Medicines Agency) now endorse eGFR slope as a surrogate end point for clinical trials.^7^^,^S5 This recognition reinforces a central physiologic insight: trajectory instability may reflect cumulative renal stress before cross-sectional impairment becomes clinically apparent.

Slope functions as an “11th dimension” of kidney risk (Figure 2b), capturing dynamic properties of the filtration system that static heat maps cannot detect. Especially in pathophysiologic states that promote glomerular hyperfiltration such as obesity, diabetes, and hypertensive cardiorenal physiology,S9–S11^,^S14–S16 eGFR slope instability may identify high-risk individuals years before conventional thresholds signal concern.^3^^,^^8^^,^S3

Importantly, slope-based assessment is not limited to hyperfiltration-related injury. Rapid eGFR decline may reflect ischemic or nephron-loss–dominant processes that often present with minimal albuminuria, suggesting that longitudinal trajectory assessment may help detect heterogeneous forms of kidney injury not fully captured by static heat-map classification. Thus, eGFR slope may serve as a unifying dynamic marker across diverse pathophysiologic pathways of kidney vulnerability within the cardiorenal continuum.

### Interpreting the High-eGFR Risk Zone in the KDIGO Heat Map

The 2024 KDIGO heat map preserves the high-eGFR/high-albuminuria risk zone identified in the CKD-PC JAMA 2023 analysis.^1^^,^^2^ Although this observation is incorporated into risk stratification, its physiologic interpretation remains implicit. Why should individuals with “supra-normal” eGFR, often ≥ 105 ml/min per 1.73 m^2^, experience cardiovascular and mortality risks exceeding those of stage 3b CKD?

Viewed through the combined lenses of hyperfiltration and eGFR slope, this pattern becomes physiologically coherent. Very high filtration may reflect glomerular hypertrophy, increased intraglomerular pressure, and tubular metabolic overload rather than preserved renal reserve.^3^, ^4^, ^5^^,^S17 Operational considerations regarding measurement limitations and nonhyperfiltration causes of elevated eGFR (e.g., reduced muscle mass) are discussed in the Supplementary Methods. Longitudinal trajectory further refines this interpretation: individuals with rapid eGFR decline or paradoxical increase experience higher risks of kidney failure, heart failure, and death regardless of baseline filtration.^7^^,^^8^ Albuminuria further signals podocyte and endothelial injury, amplifying vulnerability when superimposed on hyperfiltration.^4^^,^^5^

From this perspective, the high-eGFR/high-albuminuria region of the KDIGO heat map can be understood not as a paradox, but as the cross-sectional expression of a dynamic trajectory characterized by early filtration stress, structural remodeling, and eventual decline. What appears as a static “red zone” may therefore represent the visible surface of evolving nephron stress within the cardiorenal continuum, often beginning years or even decades before conventional CKD thresholds are crossed.

### Implications for Clinical Care and Future Guidelines

Recognizing hyperfiltration and slope instability as early manifestations of kidney stress reframes multiple aspects of CKD care.^3^, ^4^, ^5^ Rather than replacing existing staging systems, integrating dynamic filtration physiology complements KDIGO’s risk-based paradigm and may represent a natural next step in guideline evolution. eGFR values that appear reassuring may, in the presence of albuminuria or metabolic stress, reflect early maladaptive physiology rather than preserved renal reserve.

In this context, hyperfiltration can be understood as physiologic overshoot within the cardiorenal continuum. Combining hyperfiltration physiology with longitudinal eGFR trajectories enables earlier identification of individuals entering high-risk pathways, including obesity-related nephropathy, diabetic kidney disease, and tubular-glomerular mismatch states.

Current CKD staging systems excel at describing filtration loss but provide limited resolution in the pre-CKD phase. A next-generation heat-map framework integrating baseline eGFR, albuminuria, hyperfiltration physiology, and eGFR slope could enable earlier and more individualized preventive strategies. This approach remains fully aligned with KDIGO’s emphasis on risk-based care while enhancing physiologic interpretability across the lifetime filtration trajectory.

Hyperfiltration and slope are both measurable and modifiable. Therapies that reduce intraglomerular pressure and metabolic workload, including sodium-glucose cotransporter-2 inhibitors, renin-angiotensin system blockade, glucagon-like peptide-1 receptor agonists, nonsteroidal mineralocorticoid receptor antagonists, dietary interventions, and weight reduction have been shown to stabilize kidney-function trajectories.^4^^,^^5^^,^S18–S22 Regulatory recognition of eGFR slope as a surrogate end point further underscores its translational relevance.^7^

A unified heat-map framework incorporating these dynamic dimensions could support earlier population screening,S23^,^S24 more timely therapeutic initiation,S18–S22 improved prognostic communication,S25^,^S26 and more efficient allocation of health care resources,S27 in alignment with global kidney-health priorities outlined by KDIGO and the ISN, and may help bridge static risk classification with the dynamic physiology of kidney vulnerability across the lifetime filtration trajectory.

### Conclusion

The 2023 CKD Prognosis Consortium heat map and the 2024 KDIGO guidelines established a powerful, data-driven framework for CKD risk stratification based on eGFR and albuminuria. At the same time, the persistence of elevated risk in the very-high eGFR/high-albuminuria region highlights an important conceptual gap in the physiologic interpretation of kidney risk. Hyperfiltration and eGFR slope provide complementary physiologic and temporal dimensions that help bridge this gap.

Hyperfiltration, particularly in the presence of albuminuria and cardiometabolic stress, may represent early cardiorenal overload rather than preserved kidney function. Structural correlates such as glomerular hypertrophy quantified by increases in maximal glomerular diameter, reinforce the notion that “supra-normal” filtration often reflects a fragile, maladaptive state with a high likelihood of subsequent decline. In parallel, eGFR slope captures instability over time, linking filtration dynamics to kidney failure and cardiovascular outcomes and aligning CKD care with endpoints already recognized by regulatory agencies.

Reframing the KDIGO heat map within a broader dynamic framework that integrates hyperfiltration physiology, longitudinal eGFR trajectory, and patient attributes may improve the recognition of early high-risk pathways, support timely initiation of renoprotective and cardioprotective therapies, and generate testable hypotheses for future cohort studies and intervention trials. Ultimately, a next-generation CKD heat map that incorporates both the level of filtration and the trajectory of filtration change may better reflect real-world kidney risk than any static snapshot alone.

In clinical practice, very high eGFR in the presence of albuminuria or cardiometabolic stress should be interpreted as a potential warning signal rather than reassurance, and longitudinal changes in eGFR should be evaluated alongside patient attributes that modulate cardiorenal risk. For guideline development, these concepts point toward a more physiologically coherent and prevention-oriented approach to CKD staging. Hyperfiltration and eGFR slope are measurable, modifiable, and mechanistically grounded, making them natural candidates for integration into future CKD risk frameworks.

From this perspective, a next-generation CKD heat map would describe not only how much filtration remains, but how the filtration system behaves over time.

## Disclosure

All the authors declared no competing interests.

## Supplementary Material

Supplementary File (PDF) **Supplementary Abstract.** An expanded, PubMed-style summary detailing the conceptual framework, clinical implications, and research agenda related to hyperfiltration and eGFR slope. **Supplementary Translational Statement.** A clinician- and policymaker-focused explanation of how integrating hyperfiltration physiology and eGFR slope into kidney-risk assessment could influence screening strategies, therapeutic timing, and health-system planning. **Supplementary Methods.** A narrative description of the literature search strategy, inclusion criteria for key mechanistic and epidemiologic studies, and the rationale for reference selection. **Supplementary References.** A full list of supporting citations (S1–S27) corresponding to supplementary references cited throughout the manuscript, including cohort analyses of hyperfiltration, trajectory studies, mechanistic reviews, and prior work on attribute-based medicine and glomerular structure.

## Footnotes

Supplementary File (PDF)
**Supplementary Abstract.** An expanded, PubMed-style summary detailing the conceptual framework, clinical implications, and research agenda related to hyperfiltration and eGFR slope. **Supplementary Translational Statement.** A clinician- and policymaker-focused explanation of how integrating hyperfiltration physiology and eGFR slope into kidney-risk assessment could influence screening strategies, therapeutic timing, and health-system planning. **Supplementary Methods.** A narrative description of the literature search strategy, inclusion criteria for key mechanistic and epidemiologic studies, and the rationale for reference selection. **Supplementary References.** A full list of supporting citations (S1–S27) corresponding to supplementary references cited throughout the manuscript, including cohort analyses of hyperfiltration, trajectory studies, mechanistic reviews, and prior work on attribute-based medicine and glomerular structure.
