# Supplementary material for: The Next-Generation CKD Heat Map: Hyperfiltration and eGFR Slope in the Cardiorenal Continuum
Source: Kidney Int Rep. 2026 Mar 17;11(5):106486. doi: 10.1016/j.ekir.2026.106486 (PMC13088956; doi:10.1016/j.ekir.2026.106486)
Supplement: Supplementary File (PDF) — Supplementary Abstract. An expanded, PubMed-style summary detailing the conceptual framework, clinical implications, and research agenda related to hyperfiltration and eGFR slope. Supplementary Translational Statement. A clinician- and policymaker-focused explanation of how integrating hyperfiltration physiology and eGFR slope into kidney-risk assessment could influence screening strategies, therapeutic timing, and health-system planning. Supplementary Methods. A narrative description of the literature search strategy, inclusion criteria for key mechanistic and epidemiologic studies, and the rationale for reference selection. Supplementary References. A full list of supporting citations (S1–S27) corresponding to supplementary references cited throughout the manuscript, including cohort analyses of hyperfiltration, trajectory studies, mechanistic reviews, and prior work on attribute-based medicine and glomerular structure. [file mmc1.pdf]

Supplementary Material

- **Supplementary Abstract**
- **Supplementary Translational Statement**
- **Supplementary Methods**
- **Supplementary References**
- **SANRA Checklist**

Supplementary Abstract

This Supplementary Material provides operational and conceptual clarification supporting interpretation of hyperfiltration and eGFR trajectory within the cardiorenal continuum. Hyperfiltration is framed as a context-dependent clinical phenotype reflecting increased nephron workload rather than a diagnostic entity, with attention to age-dependent variation in filtration levels that distinguishes physiologic high eGFR from maladaptive filtration states.

Longitudinal eGFR slope is presented as a dynamic marker of filtration instability, encompassing both accelerated decline and paradoxical rise in eGFR. Regulatory recognition of slope as a surrogate endpoint reinforces its value as an indicator of cumulative renal stress preceding overt CKD.

By clarifying the physiologic interpretation and clinical context of these dynamic markers, this Supplementary Material provides the conceptual and operational foundation for extending the CKD heat-map framework beyond static staging. Together, these concepts support a trajectory-based interpretation of kidney risk that complements cross-sectional CKD staging and informs the proposed extension of the CKD heat-map framework.

Supplemental Translational Statement

Very early kidney injury is often difficult to detect because current risk tools focus mainly on reduced filtration. Two measurable signals can identify risk far earlier: glomerular hyperfiltration and eGFR slope. Hyperfiltration—often seen in individuals with obesity, diabetes, or hypertension—reflects increased workload on each nephron and is associated with faster decline and higher cardiovascular risk. eGFR slope captures whether kidney function is rising or falling over time, signaling instability even when eGFR appears normal. Together, these dynamic markers provide a physiologically coherent view of early cardiorenal stress beyond static classification based solely on eGFR and albuminuria. Integrating hyperfiltration and slope into the KDIGO heat map offers a practical and biologically grounded way to detect risk earlier, guide preventive therapy, and support shared decision-making in both specialist and primary care settings.

Supplementary Methods

Operational Definition of Hyperfiltration

For clinical interpretation, hyperfiltration should be considered a context-dependent clinical phenotype rather than a diagnostic entity. Hyperfiltration may be suspected when the following conditions are present:

(i) estimated glomerular filtration rate (eGFR) is persistently elevated relative to age- and sex-expected distributions;

(ii) elevated values are confirmed on repeat measurements over time;

(iii) albuminuria and/or cardiometabolic stressors (e.g., obesity, diabetes, hypertension) are present.

Before interpreting elevated eGFR as hyperfiltration, alternative explanations for apparently high eGFR should be considered, including low muscle mass, frailty, cachexia, chronic illness, or creatinine-generation bias. Because estimation equations are less precise at higher filtration ranges, eGFR should be interpreted as a clinical approximation of filtration status rather than a direct measure of glomerular filtration rate (Inker et al., PMID: 22762315).

When clinical uncertainty exists, interpretation should prioritize longitudinal trajectory, repeat measurements, and overall clinical context rather than reliance on a single eGFR value.

Age-dependent interpretation is essential. Population data indicate that median eGFR values near 105 mL/min/1.73 m² may be physiologic in younger adults (Yang et al., PMID: 41543464) but may represent supraphysiologic filtration in middle-aged populations when accompanied by albuminuria or cardiometabolic stress. Even in younger adults (<40 years), persistently elevated eGFR values accompanied by albuminuria or cardiometabolic stress may indicate increased nephron workload rather than physiologic filtration reserve (Hanai et al., PMID: 41475080).

In this Editorial, hyperfiltration is therefore conceptualized as evidence of increased nephron workload along the lifetime filtration trajectory rather than as a standalone diagnostic category.

Age-dependent interpretation of high eGFR

Interpretation of elevated eGFR requires consideration of age-related physiologic variation in filtration levels. Median eGFR values near 105 mL/min/1.73 m² are commonly observed in younger adults and decline with aging (Yang et al., PMID: 41543464).

For practical interpretation, three broad clinical strata may be considered:

• <40 years: high eGFR often physiologic, unless accompanied by albuminuria or cardiometabolic stress

• 40–65 years: persistent eGFR ≥105 mL/min/1.73 m² may suggest hyperfiltration, particularly with albuminuria or cardiometabolic stress

• ≥65 years: eGFR values ≥90 mL/min/1.73 m² may represent relative hyperfiltration

In women, physiologic transitions related to menopause (typically around age 50) and increased longevity may further influence interpretation of filtration levels; thus, age strata such as <50, 50–75, and ≥75 years may provide additional clinical context when interpreting filtration physiology in women, although age-based interpretation remains the primary framework (Leone et al., PMID: 40378426). This age-stratified framework is intended as a pragmatic clinical interpretation aid rather than a diagnostic classification.

Literature Identification and Search Strategy

This Editorial is based on a narrative synthesis of mechanistic, epidemiologic, and regulatory literature related to chronic kidney disease (CKD) risk stratification, glomerular hyperfiltration, and longitudinal changes in estimated glomerular filtration rate (eGFR). Relevant studies were identified through targeted searches of PubMed/MEDLINE and Google Scholar, complemented by manual review of reference lists from key consensus documents and landmark cohort studies. Search terms included combinations of: "glomerular hyperfiltration," "eGFR slope," "kidney function trajectory," "albuminuria," "cardiorenal outcomes," "KDIGO," "CKD Prognosis Consortium," and "surrogate endpoints." No restrictions were placed on study design; observational cohorts, mechanistic studies, clinical trials, meta-analyses, and guideline or regulatory documents were all considered.

Rationale for Reference Selection

Primary references cited in the main text were selected to represent pivotal evidence defining the prognostic significance of hyperfiltration, eGFR slope, and albuminuria across kidney and cardiovascular outcomes. These included large individual-participant meta-analyses, longitudinal cohort studies with extended follow-up, and consensus or guideline documents with direct relevance to clinical practice. Supplementary references were used to support mechanistic insights, provide additional epidemiologic confirmation, or expand on policy- and communication-related implications that could not be accommodated within the word limits of the main Editorial. This two-tiered referencing strategy was intended to preserve clarity in the main narrative while maintaining transparency and depth in the supporting evidence base.

Conceptual Framework Development

This Supplement outlines a trajectory-based physiologic framework linking hyperfiltration, eGFR slope, and lifetime kidney risk. Rather than redefining CKD staging, this framework is intended to complement existing heat-map–based risk stratification by incorporating temporal dynamics of kidney function.

Hyperfiltration represents an early filtration-load state, whereas eGFR slope reflects dynamic

instability in filtration capacity over time. Together, these dimensions describe a continuum from early nephron workload to structural exhaustion. Within this framework, cross-sectional severity and longitudinal trajectory are understood as complementary dimensions of kidney risk rather than competing models of disease progression.

Interpretation of eGFR Slope and Filtration Instability

eGFR slope was considered as a continuous measure of kidney function change over time, encompassing both rapid decline and paradoxical rise in filtration. For conceptual interpretation in this Editorial, the term "filtration instability" is used to describe sustained or unexpected deviation in longitudinal eGFR trajectory, including both accelerated decline and paradoxical rise in eGFR over time.

Evidence supporting slope as a surrogate endpoint was drawn from meta-analyses and regulatory workshops involving the US Food and Drug Administration and the European Medicines Agency. These sources collectively indicate that slope captures cumulative renal stress and injury earlier than cross-sectional thresholds and predicts kidney failure, cardiovascular events, and mortality independently of baseline eGFR and albuminuria.

This terminology is intended as a physiologic interpretation framework rather than a diagnostic classification and should be understood as complementary to existing CKD staging approaches.

Clinical, Translational, and Policy Considerations

The translational relevance of hyperfiltration and eGFR slope was assessed by reviewing evidence on the effects of established therapies—such as renin–angiotensin system blockade, sodium–glucose cotransporter 2 inhibitors, glucagon-like peptide-1 receptor agonists, nonsteroidal mineralocorticoid receptor antagonists, and lifestyle interventions—on intraglomerular hemodynamics and longitudinal kidney function trajectories. Policy-oriented literature from KDIGO, the International Society of Nephrology, and global public health organizations was reviewed to contextualize how enhanced risk stratification could inform screening strategies, prognostic communication, shared decision making, and allocation of healthcare resources.

Scope and Limitations

As an Editorial, this work does not present new empirical data and does not follow a systematic review methodology. Instead, it aims to synthesize and interpret existing evidence to provide a coherent physiologic and longitudinal framework for understanding CKD risk. While the literature selection was necessarily selective, emphasis was placed on consistency across independent cohorts, biological plausibility, and relevance to contemporary clinical and policy debates.

Supplementary References

S1. Lou-Meda R, Perez JB. Reducing the burden of chronic kidney disease in the world. *Lancet*. 2025;405:1810.

S2. Wen WL, Lee YJ, Hwu DW, et al. Age- and gender-adjusted estimated glomerular filtration rate definition reveals hyperfiltration as a risk factor for renal function deterioration in type 2 diabetes. *Diabetes Obes Metab*. 2024;26:1636-1643.

S3. Turin TC, Coresh J, Tonelli M, et al. Change in the estimated glomerular filtration rate over time and risk of all-cause mortality. *Kidney Int*. 2013;83:684-691.

S4. Julian MT, Codina P, Lupon J, et al. Long-term trajectory of estimated glomerular filtration rate in ambulatory patients with type 2 diabetes and heart failure: clinical insights and prognostic implications. *Cardiovasc Diabetol*. 2025;24:104.

S5. Inker LA, Collier W, Greene T, et al. A meta-analysis of GFR slope as a surrogate endpoint for kidney failure. *Nat Med*. 2023;29:1867-1876.

S6. Oulhaj A, Aziz F, Suliman A, et al. Estimated glomerular filtration rate slope and risk of primary and secondary major adverse cardiovascular events and heart failure hospitalization in people with type 2 diabetes: An analysis of the EXSCEL trial. *Diabetes Obes Metab*. 2024;26:4602-4612.

S7. Coresh J, Turin TC, Matsushita K, et al. Decline in estimated glomerular filtration rate and subsequent risk of end-stage renal disease and mortality. *Jama*. 2014;311:2518-2531.

S8. Kataoka H, Moriyama T, Manabe S, et al. Maximum Glomerular Diameter and Oxford MEST-C Score in IgA Nephropathy: The Significance of Time-Series Changes in Pseudo-R(2) Values in Relation to Renal Outcomes. *J Clin Med*. 2019;8.

S9. Chung SM, Jung I, Lee DY, et al. Effect of Glomerular Hyperfiltration on Incident Cardiovascular Disease in Patients with Type 2 Diabetes Mellitus. *Clin J Am Soc Nephrol*. 2025;20:410-419.

S10. Dupuis ME, Nadeau-Fredette AC, Madore F, et al. Association of Glomerular Hyperfiltration and Cardiovascular Risk in Middle-Aged Healthy Individuals. *JAMA Netw Open*. 2020;3:e202377.

S11. Penno G, Orsi E, Solini A, et al. Renal hyperfiltration is independently associated with increased all-cause mortality in individuals with type 2 diabetes: a prospective cohort study. *BMJ Open Diabetes Res Care*. 2020;8.

S12. Park M, Yoon E, Lim YH, et al. Renal hyperfiltration as a novel marker of all-cause mortality. *J Am Soc Nephrol*. 2015;26:1426-1433.

S13. Thomas M, Lemaitre M, Wilson ML, et al. Applications of Extreme Value Theory in

Public Health. *PLoS One*. 2016;11:e0159312.

S14. Magee GM, Bilous RW, Cardwell CR, et al. Is hyperfiltration associated with the future risk of developing diabetic nephropathy? A meta-analysis. *Diabetologia*. 2009;52:691-697.

S15. Melsom T, Brobak KM, Norvik JV, et al. Iohexol clearance, but not estimated GFR, reveals a steeper GFR decline in patients with prediabetes. *Kidney Int*. 2025.

S16. Eriksen BO, Lochen ML, Arntzen KA, et al. Subclinical cardiovascular disease is associated with a high glomerular filtration rate in the nondiabetic general population. *Kidney Int*. 2014;86:146-153.

S17. Kanbay M, Copur S, Guldan M, et al. Proximal tubule hypertrophy and hyperfunction: a novel pathophysiological feature in disease states. *Clin Kidney J*. 2024;17:sfae195.

S18. Colhoun HM, Lingvay I, Brown PM, et al. Long-term kidney outcomes of semaglutide in obesity and cardiovascular disease in the SELECT trial. *Nat Med*. 2024;30:2058-2066.

S19. Perkovic V, Jardine M, Neal B, et al. Canagliflozin and Renal Outcomes in Type 2 Diabetes and Nephropathy. *The New England Journal of Medicine*. 2019;380:2295-2306.

S20. The E-KCG, Herrington WG, Staplin N, et al. Empagliflozin in Patients with Chronic Kidney Disease. *N Engl J Med*. 2023;388:117-127.

S21. Bakris G, Agarwal R, Anker S, et al. Effect of Finerenone on Chronic Kidney Disease Outcomes in Type 2 Diabetes. *The New England Journal of Medicine*. 2020;383:2219-2229.

S22. Heerspink HJL, Jongs N, Chertow GM, et al. Effect of dapagliflozin on the rate of decline in kidney function in patients with chronic kidney disease with and without type 2 diabetes: a prespecified analysis from the DAPA-CKD trial. *Lancet Diabetes Endocrinol*. 2021;9:743-754.

S23. Kataoka H, Hayashi T, Nangaku M, et al. Attribute based cross classification analyses from the BRIGHTEN study reveal that therapeutic responsiveness to erythropoiesis stimulating agents predicts cardiorenal prognosis in renal anemia. *Sci Rep*. 2025;15:23221.

S24. Kataoka H, Manabe S, Moriyama T, et al. Attribute-Based Medicine for IgA Nephropathy: Risk Factor Constellations Influence Kidney Prognosis. *Nephrol Dial Transplant*. 2025.

S25. Levey AS, Eckardt KU, Dorman NM, et al. Nomenclature for kidney function and disease: report of a Kidney Disease: Improving Global Outcomes (KDIGO) Consensus Conference. *Kidney Int*. 2020;97:1117-1129.

S26. Elwyn G, Frosch D, Thomson R, et al. Shared decision making: a model for clinical practice. *J Gen Intern Med*. 2012;27:1361-1367.

S27. Luyckx VA, Tonelli M, Stanifer JW. The global burden of kidney disease and the sustainable development goals. *Bull World Health Organ*. 2018;96:414-422D.

SANRA Checklist

Manuscript Title: ***The Next-Generation CKD Heat Map: Hyperfiltration and eGFR Slope in the Cardiorenal Continuum***
Manuscript Type: Editorial (*Kidney International Reports*)
Authors: Hiroshi Kataoka, Yusuke Ushio, Shun Manabe, Junichi Hoshino

1. Explanation of the importance of the review (Aim of the article)
✓ Yes — This manuscript clearly defines the major knowledge gap in current KDIGO 2024 CKD risk stratification, highlighting the absence of hyperfiltration and eGFR slope despite their strong prognostic value. The aim—to propose a next-generation dynamic CKD heat map integrating these dimensions—is explicitly stated and clinically impactful.
2. Literature search described
✓ Yes — The manuscript draws upon multiple high-quality meta-analyses (e.g., CKD-PC 2023, JAMA 2023), landmark cohort studies, and mechanistic literature. Although not a systematic review, the selection of evidence is transparently grounded in recent epidemiology, KDIGO 2024 guideline content, and regulatory documents validating eGFR slope as a surrogate endpoint.
3. Referencing
✓ Yes — References include major peer-reviewed journals (JAMA, Kidney Int, Lancet, JASN) and guideline documents. Citation structure is consistent with KI guidelines and supports all key mechanistic and epidemiologic claims.
4. Scientific reasoning (Logical structure of arguments)
✓ Yes — The manuscript constructs a coherent argument:
(1) Hyperfiltration represents a systemic risk phenotype →
(2) Alters lifetime filtration trajectory → early burnout →
(3) eGFR slope reflects dynamic instability →
(4) Integrating both yields a next-generation heat map.
The logic is internally consistent and supported by evidence.
5. Appropriate data presentation
✓ Yes — Although no new data are presented, the manuscript uses conceptual figures (KDIGO-derived heat map, trajectory models, slope-based risk concept) that are appropriate for an Editorial.

The figures faithfully illustrate mechanistic and epidemiologic concepts.

6. Writing quality

✓ Yes — The manuscript is clearly written, succinct, and accessible to multidisciplinary readers (nephrology, cardiology, endocrinology, primary care). Argumentation is balanced, terminology is consistent, and the structure follows KI expectations for Editorial articles.

Final SANRA Checklist Statement

This manuscript meets all SANRA criteria. It is a narrative, conceptual review integrating epidemiologic, mechanistic, and regulatory evidence to propose a next-generation CKD heat map for early cardiorenal risk detection.
